# Supplementary material for: Metagenomics-Based Proficiency Test of Smoked Salmon Spiked with a Mock Community
Source: Microorganisms. 2020 Nov 25;8(12):1861. doi: 10.3390/microorganisms8121861 (PMC7760972; doi:10.3390/microorganisms8121861)
Supplement: Supplementary file 1 [file microorganisms-08-01861-s001.pdf]

Metagenomics-based proficiency test of smoked salmon spiked with a mock community

Claudia Sala[1], Hanne Mordhorst[2], Josephine Grützke[3], Annika Brinkmann[4], Thomas Petersen[2], Casper Poulsen[2], Paul D. Cotter[5], Fiona Crispie[5], Richard J. Ellis[6], Gastone Castellani[7], Clara Amid[8], Mikhayil Hakhverdyan[9], Françoise S. Le Guyader[10], Gerardo Manfreda[11], Joel Mossong[12], Andreas Nitsche[4], Catherine Ragimbeau[12], Julien Schaeffer[10], Joergen Schlundt[13], Moon Y. F. Tay[13], Frank M. Aarestrup[2], Rene Hendriksen[2], Sünje Johanna Pamp[2], Alessandra De Cesare[14*]

*alesandra.decesare@unibo.it

Supplementary Figure S1. Within-DNA workflow log-variance of the detected abundances. Box plots show the distribution of the variances observed in each workflow considering all the mock community microorganisms. Red dots indicate the variance observed for the specific microorganism indicated in the subplot title. For WF12 used for one metagenome, box plots are not shown, and the red dot is drawn on the x-axis to indicate that the variance is not defined. For each microorganism, we used the average of the variances observed in all workflows with at least two samples as estimation of the variance of the same microorganism in WF12 including one sample only.

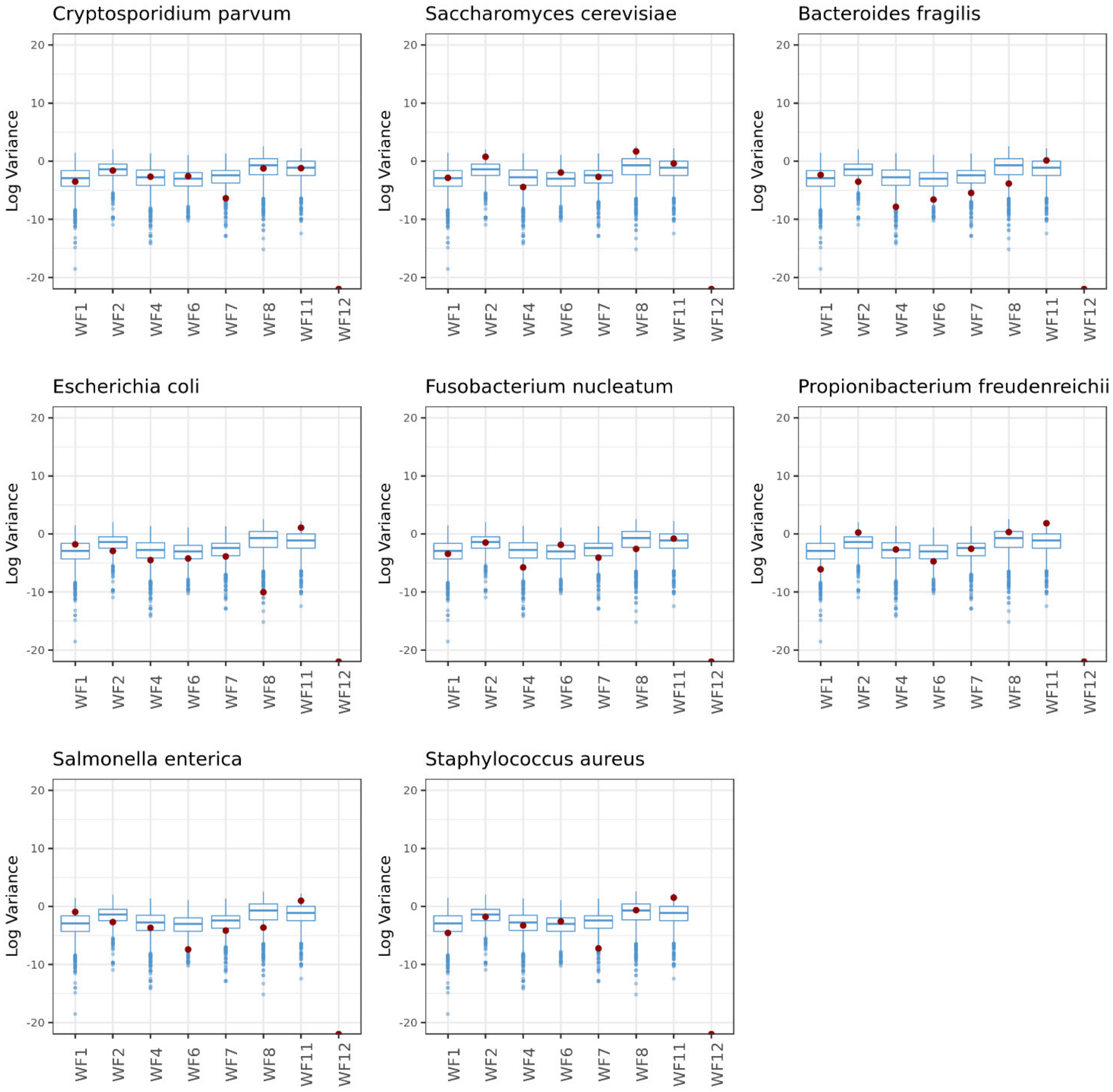

Supplementary Figure S2. Box plots of the DESeq2 normalized read counts (detected abundances) obtained in group of samples defined by: a) the categorized pre-processing procedure (i.e., categorized as protocols based on a beating protocol with TissueLyser (BBTL), no-processing (NO_PP) and other pre-processing protocols (OTHER_PP); b) the DNA extraction protocol (i.e. DNeasy Power Food Microbial Kit (PowerFood), QIAamp DNA Mini Kit with or without SISPA and QIAamp UCP Pathogen Mini Kit, categorized as QIAamp, or the QIAamp Fast DNA Stool and DNeasy Power Soil, categorized as OTHER-EXD; c) the library preparation strategy (i.e., Nextera™ XT DNA Library Prep kit and the Nextera™ DNA Flex Library Preparation kit, categorized as NexteraXT, and the NEBNext® Ultra™ II DNA Library Prep Kit for Illumina®, TruSeq® DNA Library Prep Kit, GeneRead DNA Library kit other strategies, categorized as OTHER_L); d) the sequencing platform (i.e., NextSeq500 and the HiSeq2500, categorized as NextSeq500, and the MiniSeq, MiSeq and Ion Torrent S5XL, categorized as OTHER_SP). Plots for the other steps of the workflow are not shown because no statistically significant difference was observed. For each variable, plots are drawn separately for each mock community microorganism. In the figure, dots represent the values detected in each individual DNA metagenome, and a "*" symbol is added to the species name (x-axis) if the average abundances of at least two sample groups were statistically significantly different.

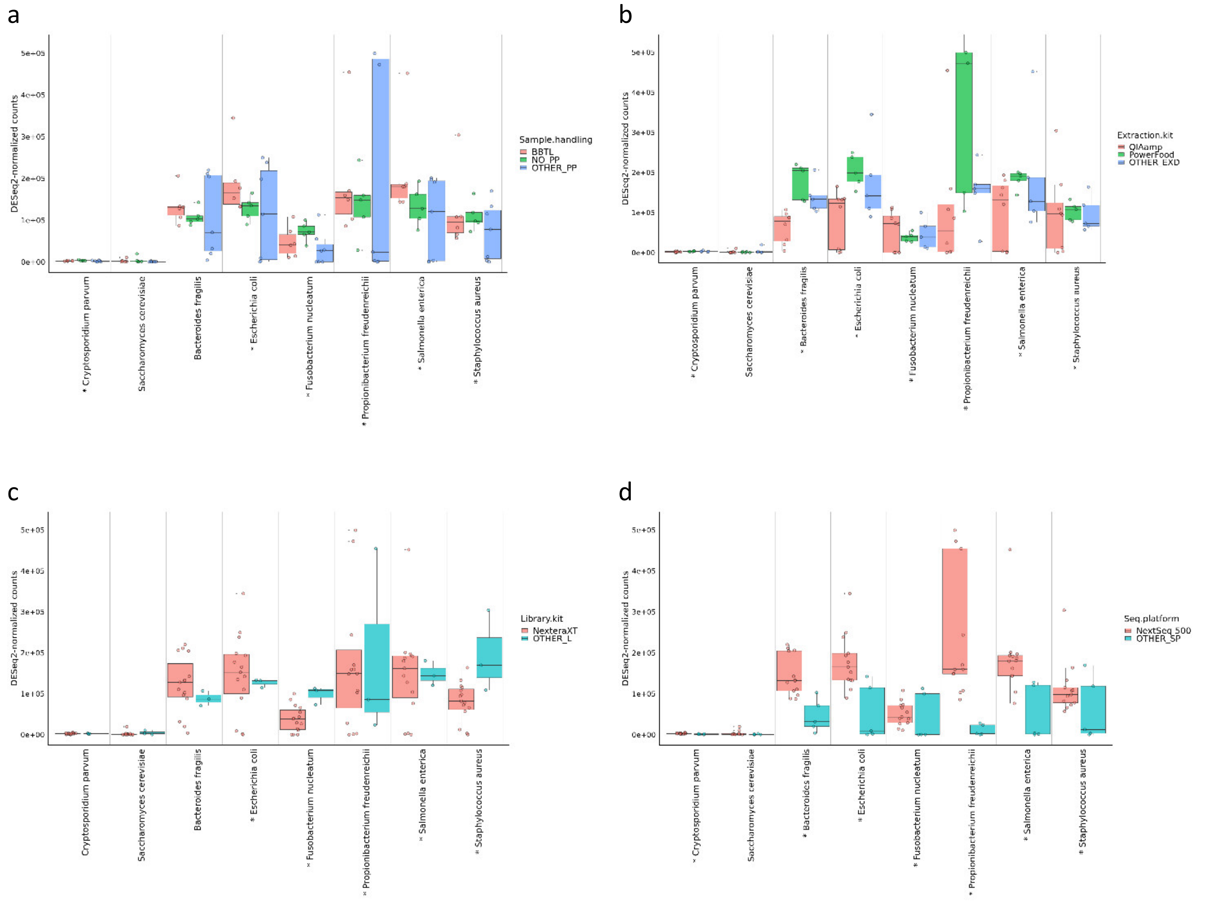

Supplementary Figure S3. Box plots of the mock community microorganisms' abundances (DESeq2 normalized counts) detected in the DNA workflows. Blue dots indicate the values of the samples included in each workflow. For WF12 including only one sample, the box plot is substituted with a horizontal line that overlaps the blue dot relative to that sample. Each subplot refers to one of the mock community microorganisms, as indicated in the title. Viruses were not included in this analysis.

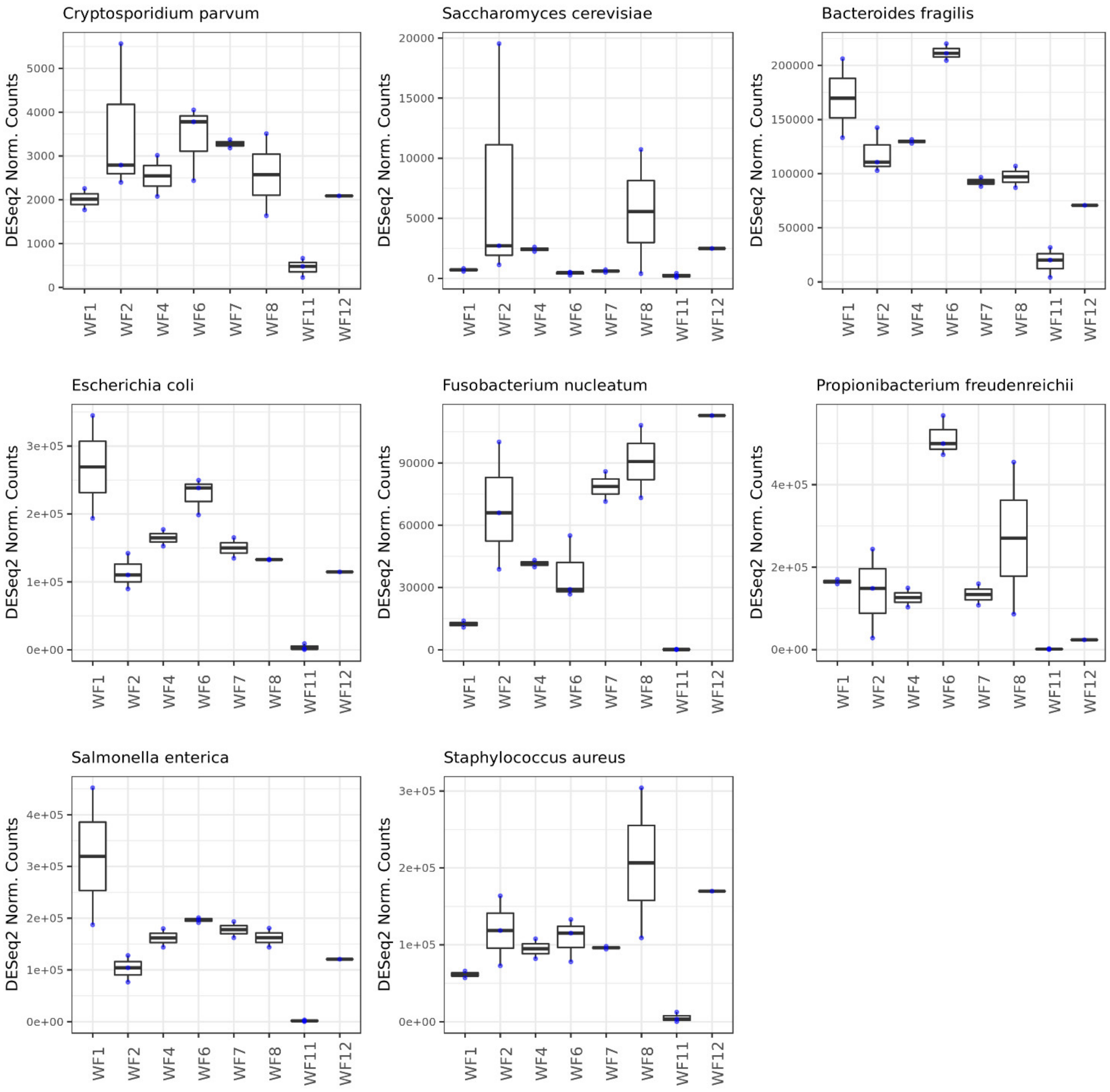

Supplementary Table S1. Relative abundance of the microorganisms of the mock community: expected values and relative abundance quantified in the DNA metagenomic datasets.

Species	Expected	M06	M07	M08	M10	M11	M13	M15	M16	M18
Bacteroides fragilis	0.065	0.164	0.131	0.530	0.121	0.696	0.739	0.178	0.182	0.176
Bovine alphaherpesvirus 1	0.081	0.002	0.002	0.000	0.003	0.000	0.000	0.002	0.002	0.002
Cryptosporidium parvum	0.002	0.001	0.004	0.004	0.005	0.016	0.118	0.003	0.004	0.003
Escherichia coli	0.064	0.275	0.182	0.153	0.226	0.063	0.053	0.259	0.217	0.237
Fusobacterium nucleatum	0.027	0.009	0.116	0.004	0.098	0.004	0.012	0.019	0.057	0.058
Propionibacterium freudenreichii	0.331	0.136	0.216	0.043	0.147	0.042	0.004	0.213	0.213	0.138
Saccharomyces cerevisiae	0.015	0.001	0.001	0.003	0.001	0.015	0.015	0.001	0.004	0.003
Salmonella enterica	0.062	0.360	0.220	0.055	0.265	0.050	0.025	0.250	0.205	0.240
Staphylococcus aureus	0.352	0.053	0.128	0.207	0.134	0.113	0.034	0.076	0.117	0.144

Species	Expected	M24	M26	M27	M29	M30	M31	M32	M36	M38
Bacteroides fragilis	0.065	0.187	0.130	0.115	0.165	0.144	0.174	0.173	0.084	0.173
Bovine alphaherpesvirus 1	0.081	0.001	0.003	0.000	0.001	0.001	0.001	0.001	0.001	0.001
Cryptosporidium parvum	0.002	0.007	0.002	0.003	0.004	0.003	0.002	0.003	0.003	0.004
Escherichia coli	0.064	0.145	0.197	0.186	0.228	0.176	0.163	0.188	0.105	0.141
Fusobacterium nucleatum	0.027	0.087	0.161	0.183	0.161	0.039	0.024	0.021	0.058	0.061
Propionibacterium freudenreichii	0.331	0.195	0.128	0.039	0.045	0.401	0.411	0.372	0.358	0.382
Saccharomyces cerevisiae	0.015	0.026	0.001	0.004	0.002	0.000	0.000	0.000	0.008	0.004
Salmonella enterica	0.062	0.137	0.215	0.196	0.205	0.142	0.162	0.151	0.142	0.120
Staphylococcus aureus	0.352	0.215	0.163	0.275	0.190	0.094	0.064	0.091	0.240	0.114

Supplementary Table S2. Read counts of the microorganisms of the mock community in the tested metagenomes. The target nucleic acid is specified under the metagenomic dataset label.

Taxa	**M06** (DNA)	**M07** (DNA)	**M08** (DNA)	**M10** (DNA)	**M11** (DNA)	**M13** (DNA)	**M15** (DNA)	**M16** (DNA)	**M18** (DNA)	**M24** (DNA)	**M26** (DNA)	**M27** (DNA)	**M29** (DNA)	**M30** (DNA)
Cryptosporidium parvum	1234	1591	77	1796	62	1250	1915	2130	1890	10049	3250	695	1654	5920
Saccharomyces cerevisiae	578	249	67	383	55	154	501	1842	2037	35282	797	828	777	811
Border disease virus	0	0	0	0	0	0	0	0	0	0	0	0	0	0
Bovine alphaherpesvirus 1	1750	733	0	1274	0	0	1286	826	1695	733	3490	25	233	1543
Bovine viral diarrhea virus 1	0	0	0	0	0	0	0	0	0	0	0	0	0	0
Bacteroides fragilis	143880	48336	10824	46933	2620	7835	113068	90402	119796	257443	173162	23552	70950	320353
Escherichia coli	240680	67403	3129	87984	237	558	164242	107810	161271	199039	262927	38177	98103	390957
Fusobacterium nucleatum	7539	42944	88	38038	15	129	11836	28162	39282	119134	215042	37549	69097	86149
Propionibacterium freudenreichii	118847	79848	889	57298	157	45	135097	105545	93813	268228	171049	7911	19395	889004
Salmonella enterica	315430	81094	1127	103041	190	269	158809	101602	163738	188021	286082	40171	88319	314740
Staphylococcus aureus	45996	47189	4239	52198	427	360	48404	57904	98186	295697	216801	56512	81869	208262

Taxa	**M31** (DNA)	**M32** (DNA)	**M36** (DNA)	**M38** (DNA)	**M12** (RNA)	**M19** (RNA)	**M20** (RNA)	**M23** (RNA)	**M25** (RNA)	**M28** (RNA)	**M33** (RNA)	**M34** (RNA)	**M37** (RNA)
Cryptosporidium parvum	3769	8836	26023	10194	148	19	17	77	3114	3	2	2	2317
Saccharomyces cerevisiae	401	1042	79520	9919	40	27	41	40	434	115	42	23	437
Border disease virus	0	0	0	0	0	0	2	2	4	0	2	0	0
Bovine alphaherpesvirus 1	1042	1893	7392	2250	0	0	0	29	595	0	0	1	950
Bovine viral diarrhea virus 1	0	0	0	0	0	0	0	0	0	0	0	0	1
Bacteroides fragilis	326861	479968	793554	403946	10647	144	357	2313	47747	19	116	59	88453
Escherichia coli	307329	519690	989420	327497	163	73	157	4650	60314	12	1425	865	124875
Fusobacterium nucleatum	45016	58361	543021	141582	4	13	44	1407	67781	2	152	594	59999
Propionibacterium freudenreichii	773147	1030657	3370803	890096	4	443	470	7310	22612	8	70	205	40600
Salmonella enterica	305186	417369	1339955	278870	36	35	47	4521	67229	10	171	323	129018
Staphylococcus aureus	120615	251204	2255476	265639	31	72	89	1542	83967	34	176	159	64509

Supplementary Table S3. Percentage of metagenomic datasets obtained from DNA and RNA in which each microorganism of the mock community was detected. The detection of each microorganism was defined by the presence of at least 1, 5 or 10 corresponding reads enumerated in MG-RAST.

	DNA (n=18)			RNA (n=9)		
	1 read	5 reads	10 reads	1 read	5 reads	10 reads
Cryptosporidium parvum	100	100	100	100	67	67
Saccharomyces cerevisiae	100	100	100	100	100	100
Border disease virus	0	0	0	44	0	0
Bovine alphaherpesvirus 1	83	83	83	56	33	33
Bovine viral diarrhea virus 1	0	0	0	11	0	0
Bacteroides fragilis	100	100	100	100	100	100
Escherichia coli	100	100	100	100	100	100
Fusobacterium nucleatum	100	100	100	100	78	78
Propionibacterium freudenreichii	100	100	100	100	89	78
Salmonella enterica	100	100	100	100	100	100
Staphylococcus aureus	100	100	100	100	100	100

Supplementary Table S4. Ranking of the DNA metagenomic datasets in relation to their similarity to the expected composition of the mock community considering bacteria only. The distance from the expected mock community composition is measured as Bray-Curtis dissimilarity.

Metagenome dataset	Distance from the mock community	Rank
M36	0.152	1
M38	0.275	2
M30	0.296	3
M32	0.308	4
M24	0.332	5
M31	0.332	6
M07	0.411	7
M16	0.425	8
M27	0.441	9
M26	0.464	10
M18	0.473	11
M10	0.473	12
M15	0.478	13
M29	0.520	14
M08	0.543	15
M06	0.590	16
M11	0.646	17
M13	0.779	18

Supplementary Table S5. Comparison of the mock community microorganisms' abundances detected in the DNA metagenomic datasets when applying different pre-processing protocols categorized as protocols based on a beating protocol with TissueLyser (BBTL), no-processing (NO_PP) and other pre-processing protocols (OTHER_PP). The mean abundance values are shown followed by Fold Changes (FC) and Wald's test adjusted p-value relative to the three pairwise comparisons. Following the DESeq2 notation, the Fold Change reported in the column FC_BBTL_vs_NO_PP corresponds to the ratio between the mean abundance observed applying the BBTL pre-processing over the mean abundance observed when no pre-processing (NO_PP) was applied. An analogous interpretation holds for the other columns. The multiple regression model quantifies the effect of the sample pre-processing protocol after adjustment for all the other experimental variables. Values in bold show a statistical significative difference between the compared pre-processing protocols.

Taxa	Norm_Mean BBTL	Norm_Mean NO_PP	Norm_Mean OTHER_PP	FC_BBTL_vs_NO_PP	Wald_AdjPval_BBTL_vs_NO_PP	FC_BBTL_vs_OTHER_PP	Wald_AdjPval_BBTL_vs_OTHER_PP	FC_NO_PP_vs_OTHER_PP	Wald_AdjPval_NO_PP_vs_OTHER_PP
Cryptosporidium parvum	2376.749	3461.322	1960.121	3.935	**0.013**	1.113	0.584	0.636	0.525
Saccharomyces cerevisiae	2898.220	4919.207	634.896	2.053	0.244	2.499	0.139	1.069	0.942
Bacteroides fragilis	132184.327	108151.568	108966.547	2.564	0.092	0.974	0.935	0.593	0.442
Escherichia coli	188982.042	128407.978	116081.735	4.558	**0.029**	1.030	0.953	0.630	0.449
Fusobacterium nucleatum	48188.256	72430.751	32016.463	37.906	**0.000**	13.037	**0.000**	0.640	0.482
Propionibacterium freudenreichii	187103.188	137529.573	223931.232	4.955	**0.024**	0.983	0.969	0.539	0.330
Salmonella enterica	214575.028	132820.474	102169.717	6.852	**0.011**	1.055	0.909	0.553	0.352
Staphylococcus aureus	120997.935	109495.754	73120.386	6.136	**0.013**	1.142	0.706	0.647	0.482

Supplementary Table S6. Comparison of the mock community microorganisms' abundances detected in the DNA metagenomic datasets when DNA was extracted by DNeasy Power Food Microbial Kit (PowerFood), QIAamp DNA Mini Kit with or without SISPA and QIAamp UCP Pathogen Mini Kit, categorized as QIAamp, or the QIAamp Fast DNA Stool and DNeasy Power Soil, categorized as OTHER-EXD. The mean abundance values are shown followed by the Fold Changes (FC) and Wald's test adjusted p-value relative to the 3 pairwise comparisons. Following the DESeq2 notation, the Fold Change reported in the column FC_PowerFood_vs_QIAamp corresponds to the ratio between the average abundance observed using the PowerFood extraction kit over the average abundance observed when using the QIAamp. An analogous interpretation holds for the other columns. The multiple regression model quantifies the effect of the DNA extraction kit after adjustment for all the other experimental variables. Values in bold show a statistical significative difference between the compared DNA extraction protocols.

Taxa	Norm_Mean PowerFood	Norm_Mean QIAamp	Norm_Mean OTHER_EXD	FC_Power Food_vs_ QIAamp	Wald_AdjPval_Power Food_vs_QIAamp	FC_PowerFood_v s_OTHER_EXD	Wald_AdjPval_Power Food_vs_OTHER_EXD	FC_QIAamp_vs_ OTHER_EXD	Wald_AdjPval_ QIAamp_vs_OTHER_EXD
Cryptosporidium parvum	3071.792	1893.903	2955.553	0.287	**0.007**	0.999	1.000	0.377	0.073
Saccharomyces cerevisiae	1219.965	1941.518	4959.532	0.280	0.106	1.000	1.000	0.431	0.266
Bacteroides fragilis	179087.904	63221.785	139083.167	0.218	**0.006**	1.001	1.000	0.217	**0.018**
Escherichia coli	203263.631	86459.072	176102.712	0.102	**0.002**	1.000	1.000	0.142	**0.012**
Fusobacterium nucleatum	38775.082	56490.547	45919.748	0.022	**0.000**	0.998	1.000	0.105	**0.003**
Propionibacterium freudenreichii	358471.807	104438.820	149983.205	0.103	**0.003**	1.000	1.000	0.161	**0.018**
Salmonella enterica	182652.802	100729.742	189527.723	0.072	**0.000**	1.000	1.000	0.098	**0.004**
Staphylococcus aureus	103188.485	98913.960	95610.995	0.111	**0.002**	1.000	1.000	0.204	**0.031**

Supplementary Table S7. Comparison of the mock community microorganisms' abundances detected in the DNA metagenomic datasets when using the Nextera™ XT DNA Library Prep kit and the Nextera™ DNA Flex Library Preparation kit, categorized as NexteraXT, or the NEBNext® Ultra™ II DNA Library Prep Kit for Illumina®, TruSeq® DNA Library Prep Kit, GeneRead DNA Library kit other strategies, categorized as OTHER_L. The mean abundance values are shown followed by the Fold Change (FC) and Wald's test adjusted p-value relative to the comparison. Following the DESeq2 notation, the Fold Change reported in the column FC_ NexteraXT _vs_OTHER_L corresponds to the ratio between the average abundance observed using the NexteraXT library preparation kit over the average abundance observed when using the other library preparation strategies (OTHER_L). The multiple regression model quantifies the effect of the library preparation strategy after adjustment for all the other experimental variables. Values in bold show a statistical significative difference between the compared library preparation protocols.

Taxa	Norm_Mean NexteraXT	Norm_Mean OTHER_L	FC_NexteraXT_vs_OTHER_L	Wald_AdjPval_NexteraXT_vs_OTHER_L
Cryptosporidium parvum	2537.072	2410.622	0.153	0.074
Saccharomyces cerevisiae	2187.765	4537.718	0.216	0.264
Bacteroides fragilis	122118.507	88284.009	0.123	0.077
Escherichia coli	147210.832	126780.605	0.044	**0.026**
Fusobacterium nucleatum	38748.380	98057.612	0.004	**0.000**
Propionibacterium freudenreichii	187559.779	188129.643	0.050	**0.031**
Salmonella enterica	148099.282	148417.111	0.021	**0.011**
Staphylococcus aureus	80154.830	194328.879	0.031	**0.016**

Supplementary Table S8. Comparison of the mock community microorganisms' abundances detected in the DNA metagenomic datasets when using the NextSeq500 and the HiSeq2500, categorized as NextSeq500, and the MiniSeq, MiSeq and Ion Torrent S5XL, categorized as OTHER_SP. The mean abundance values are shown followed by the Fold Change (FC) and Wald's test adjusted p-value relative to the comparison. Following the DESeq2 notation, the Fold Change reported in the column FC_NextSeq 500_vs_OTHER_SP corresponds to the ratio between the average abundance observed using the NextSeq 500 sequencing platform over the average abundance observed when using the other sequencing platforms (OTHER_SP). The multiple regression model quantifies the effect of the sequencing platform after adjustment for all the other experimental variables. Values in bold show a statistical significative difference between sequencing platforms.

Taxa	Norm_Mean NextSeq 500	Norm_Mean OTHER_SP	FC_NextSeq 500_vs_OTHER_SP	Wald_AdjPval_NextSeq 500_vs_OTHER_SP
Cryptosporidium parvum	3033.740	1169.865	3.544	**0.007**
Saccharomyces cerevisiae	3239.577	863.026	0.727	0.754
Bacteroides fragilis	143611.890	45935.012	4.641	**0.007**
Escherichia coli	178489.442	53628.309	12.402	**0.001**
Fusobacterium nucleatum	50926.123	42671.785	10.609	**0.000**
Propionibacterium freudenreichii	255544.162	11142.301	40.573	**0.000**
Salmonella enterica	185629.332	50711.850	24.023	**0.000**
Staphylococcus aureus	113921.617	60865.611	5.096	**0.032**

Supplementary Table S9. Average abundance of each microorganism of the mock community detected in the spiked samples by the workflows (WF) including DNA as target nucleic acid.

	Workflow label (n samples)							
**Taxa**	**WF1 (2)**	**WF2 (3)**	**WF4 (2)**	**WF6 (3)**	**WF7 (2)**	**WF8 (2)**	**WF11 (3)**	**WF12 (1)**
Cryptosporidium parvum	2012.741	3584.094	2545.598	3422.588	3277.164	2571.907	455.010	2088.052
Saccharomyces cerevisiae	709.401	7792.952	2422.499	418.276	608.589	5562.760	233.936	2487.636
Bacteroides fragilis	169726.919	118653.999	129779.758	211960.001	92397.922	97046.306	18708.802	70759.415
Escherichia coli	269244.414	114008.244	164880.122	228852.637	150007.579	132821.591	3771.868	114698.632
Fusobacterium nucleatum	12377.085	68281.524	41507.202	36953.669	78654.592	90680.481	147.453	112811.875
Propionibacterium freudenreichii	164772.321	140123.794	126226.647	513301.914	133638.241	270310.595	1281.716	23767.737
Salmonella enterica	319602.834	102810.983	161841.278	196527.150	177834.710	162280.973	1639.061	120689.388
Staphylococcus aureus	61482.767	118363.148	94909.789	108707.616	96194.664	206601.249	5311.904	169784.140

Supplementary Table S10. *t-test* adjusted p-values relative to the pairwise comparisons of the workflows (WF) including DNA as target nucleic acid. Values in bold show a statistical significative difference between workflows. Values in bold show a statistical significative difference between WFs.

Taxa	WF1_vs_WF2	WF1_vs_WF4	WF1_vs_WF6	WF1_vs_WF7	WF1_vs_WF8	WF1_vs_WF11	WF1_vs_WF12
Cryptosporidium parvum	0.271	0.502	0.188	0.414	0.809	0.059	0.889
Saccharomyces cerevisiae	0.271	0.227	0.207	0.671	0.807	0.108	0.326
Bacteroides fragilis	0.373	0.502	0.593	0.484	0.620	0.073	0.326
Escherichia coli	0.271	0.502	0.738	0.513	0.620	0.059	0.326
Fusobacterium nucleatum	0.143	0.227	0.134	0.090	0.187	**0.032**	0.087
Propionibacterium freudenreichii	0.529	0.502	**0.004**	0.589	0.862	0.073	0.326
Salmonella enterica	0.287	0.502	0.604	0.589	0.622	0.059	0.326
Staphylococcus aureus	0.271	0.444	0.182	0.379	0.620	0.108	0.326
**Taxa**	**WF2_vs_WF4**	**WF2_vs_WF6**	**WF2_vs_WF7**	**WF2_vs_WF8**	**WF2_vs_WF11**	**WF2_vs_WF12**	**WF4_vs_WF6**
Cryptosporidium parvum	0.649	0.998	0.948	0.656	**0.031**	0.502	0.442
Saccharomyces cerevisiae	0.705	0.191	0.335	0.772	0.066	0.841	**0.040**
Bacteroides fragilis	0.649	0.103	0.335	0.656	0.077	0.502	**0.003**
Escherichia coli	0.270	0.103	0.335	0.656	0.066	0.966	0.143
Fusobacterium nucleatum	0.584	0.222	0.747	0.656	**0.003**	0.502	0.601
Propionibacterium freudenreichii	0.783	0.194	0.822	0.656	0.066	0.502	0.143
Salmonella enterica	0.270	0.108	0.221	0.415	0.066	0.841	0.442
Staphylococcus aureus	0.705	0.962	0.747	0.656	0.077	0.841	0.608
**Taxa**	**WF4_vs_WF7**	**WF4_vs_WF8**	**WF4_vs_WF11**	**WF4_vs_WF12**	**WF6_vs_WF7**	**WF6_vs_WF8**	**WF6_vs_WF11**
Cryptosporidium parvum	0.734	0.989	0.061	0.699	0.913	0.538	**0.041**
Saccharomyces cerevisiae	0.196	0.989	0.061	0.973	0.385	0.538	0.254
Bacteroides fragilis	0.196	0.638	0.078	0.538	0.119	0.218	0.063
Escherichia coli	0.734	0.638	0.070	0.699	0.177	0.144	0.063
Fusobacterium nucleatum	0.196	0.638	**0.033**	0.489	0.177	0.218	**0.006**
Propionibacterium freudenreichii	0.896	0.989	0.077	0.538	0.177	0.538	0.063
Salmonella enterica	0.734	0.989	0.061	0.699	0.573	0.538	0.063
Staphylococcus aureus	0.896	0.924	0.087	0.699	0.678	0.538	0.093
**Taxa**	**WF6_vs_WF12**	**WF7_vs_WF8**	**WF7_vs_WF11**	**WF7_vs_WF12**	**WF8_vs_WF11**	**WF8_vs_WF12**	**WF11_vs_WF12**
Cryptosporidium parvum	0.358	0.744	0.078	0.656	0.078	0.936	0.064
Saccharomyces cerevisiae	0.330	0.744	0.126	0.656	0.370	0.936	0.104
Bacteroides fragilis	0.274	0.744	0.104	0.656	0.096	0.936	0.104
Escherichia coli	0.425	0.744	0.089	0.721	0.078	0.936	0.073
Fusobacterium nucleatum	0.184	0.744	**0.019**	0.656	**0.007**	0.936	**0.004**
Propionibacterium freudenreichii	0.274	0.744	0.090	0.656	0.078	0.863	0.104
Salmonella enterica	0.500	0.744	0.078	0.656	0.078	0.936	0.064
Staphylococcus aureus	0.545	0.744	0.104	0.656	0.078	0.936	0.084

Detailed workflows applied by the participants

Workflow 1

M06: 1 ml InhibitEX Buffer (Qiagen) was added to the frozen spiked salmon sample, and vortexed for 15 sec. The supernatant was transferred to a Power Bead Tube Garnet 0.70 tube (Oxoid). The beat-beating tube was placed on ice. *supernatant

M15: The frozen spiked salmon sample was transferred to a Power Bead Tube Garnet 0.70 tube (Oxoid) and placed on ice. To collect any remaining sample material in the original tube, 1 ml InhibitEX Buffer (Qiagen) was added to the tube that originally contained the salmon, vortexed for 10 sec, and the buffer transferred to the bead-beating tube as well. The beat-beating tube was placed on ice.

Both samples: The bead beating tubes were vortexed and inserted into the prechilled adapter of a Tissue Lyser II and bead-beating was applied for 3 x 30 sec at 30 Hz. The sample was heated at 95°C for 7 min, vortexed for 15 sec. and centrifuged at full speed for 1 min. Subsequently, the supernatant was transferred to a new 2 ml tube. DNA extraction was performed based on QIAamp Fast DNA stool mini kit (Qiagen) according to manufacturer's instruction but with 30 µl proteinase K, 400 µl supernatant, 400 µl Buffer AL and 400 µl ethanol. DNA was eluted into low-DNA bind tubes using 2 x 30 µl ATE (see laboratory protocol below). DNA underwent Nextera XT library preparation (Illumina) and the samples were sequenced on an Illumina Nextseq 500.

Workflow 2

M24: DNA was extracted from an aliquot of the spiked smoked salmon (0.25 g) using the DNeasy PowerSoil kit (Qiagen) following the manufacturer's instructions. The DNA was then diluted to 0.2 ng μl^{-1} with molecular biology grade water and 1 ng was used as the input to the NexteraXT library preparation kit (Illumina) and processed according to the manufacturer's instructions. The DNA library was then sequenced in paired-end mode with 2 × 151 cycles on the NextSeq 500 sequencing system (Illumina).

M29: The salmon sample was stored at -80°C upon arrival for 1 month before performing the total genomic DNA extraction step. The DNeasy PowerSoil kit (code 12888, Qiagen, Venlo Netherlands) was used according to the supplier's protocol without modification (no enzyme treatment). Briefly, 60 µl of C1 solution was added to the sample and the suspension containing the salmon was then transferred in the bead beating tube. Cell lysis was obtained by mechanical (10 min on vortex) and chemical methods with the different solutions provided in the kit. After applying inhibitor removal solutions, DNA was finally purified by capture on a silica membrane on a spin column and washed with ethanol based solutions as described in the standard protocol. Genomic library for metagenomic sequencing was prepared using the NexteraTM DNA Flex library prep kit (Illumina, San Diego, CA, USA). Sequencing was performed on an Illumina MiniSeq platform using a mid-output kit cartridge (FC-420-1004, Illumina, San Diego, CA, USA) to generate 2×150 bp paired-end reads.

M38: The salmon sample was stored at 4°C for <1hr on arrival, then transferred directly to a PowerBead tube from the PowerSoil kit. A 90-µl volume of 50 mg/ml lysozyme (Sigma-Aldrich, Dublin, Ireland) and 50 µl of 100 U/ml mutanolysin (Sigma-Aldrich, Dublin, Ireland) were added, and the sample was incubated at 60°C for 15 min. A 28-µl volume of proteinase K (Sigma-Aldrich, Dublin, Ireland) was added, and the sample was incubated at 60°C for a further 15 min. DNA was then purified from the sample by the standard PowerSoil DNA Isolation kit protocol (Cambio, Cambridge, United Kingdom). Following DNA isolation, the DNA was quantified using the Qubit High Sensitivity Assay (Life sciences); and a library was prepared using the Nextera XT Library prep kit (Illumina) accordingly to manufacturer's instructions. The library was sequenced on the Illumina NextSEq 500 system using a NextSeq MidOutput kit in paired end mode with 2 x 151 cycles using standard sequencing protocols (Illumina).

Workflow 3

M33-M34: Two milliliter of glycine buffer 0.05 M (pH 9) were added to the spiked salmon and homogenized in a potter grinding tube. Then the pH of was decrease to 3 by adding HCL. An equal volume of chloroform-butanol (v/v) was added and mixed by vortexing. After centrifugation the supernatant was treated with Cat-Floc T (Calgon, Ellwood City, PA) and precipitated with polyethylene glycol 6000 (PEG 6000) (Sigma, St. Quentin, France) for 1 h at 4°C and centrifuged for 20 min at 11,000 X g at 4°C. The pellet was resuspended in 2 mL of glycine buffer and filtrated using a cascade of 5, 1.2, and 0.45 mm filter pores (Minisart NML 17594, NML17593, PES16533, and PES16532). The recovered filtrates were incubated for 1 h at 37°C with 2000 Units of OmniCleave EndonucleaseTM (Lucigen corporation) and 100 mL of MgCl2 (100 mM). RNA extraction was performed using NucliSens kit bioMérieux) followed by DNase treatment for 30 min at 37°C with 25 U TURBOTM DNase (Ambion, Thermo Fisher Scientific, France). An additional RNA purification was performed using the RNA Clean & ConcentratorTM-5 kit (Zymo Research, Irvine, CA, United States). RNA extracts were transcribed into cDNA using Superscript II (Invitrogen, France) non-ribosomal hexamers (Endoh et al., 2005). Libraries were prepared using M220 Focused-ultrasonicator (Covaris) to obtained 300 pb fragment and the NEB Next Ultra DNA Library Prep Kit (New England BioLabs, France) according to the manufacturer's instructions.

Workflow 4

M16: The frozen spiked salmon sample was transferred to a Power Bead Tubes Garnet 0.70 tube (Oxoid). To collect any remaining sample material in the original tube, 500 µl Solution PF1 (55°C, solution PF1 precipitates when cold) (PowerFood Microbial DNA Isolation kit) was added to the tube that originally contained the salmon, vortexed for 10 sec, and the buffer transferred to the bead-beating tube as well. The beat-beating tube was placed on ice.

M18: 500 µl Solution PF1 (55°C) was added to the frozen spiked salmon sample and vortexed for 15 sec. The supernatant was transferred to a Power Bead Tubes Garnet 0.70 tube (Oxoid).

Both samples: The tube was inserted into the preheated (55°C) adapter of Tissue Lyser II and bead-beating was applied for 3 x 30 sec at 30 Hz. The tube was centrifuged at full speed for 1 min. Subsequently, supernatant was transferred to a new 2 ml tube. DNA extraction was performed using MoBio PowerFood Microbial DNA Isolation kit according to manufacturer's instructions using low-DNA microcentrifuge tube when eluting DNA from filter membrane. DNA underwent Nextera XT library preparation (Illumina) and the samples were sequenced on an Illumina Nextseq 500.

Workflow 5

M23: One milliliter of ice-cold buffer AL (Qiagen) was added to the frozen spiked salmon sample, transferred to a Lysing Matrix A tube (MP Biomedicals). The tube was inserted into the prechilled adaptor of Tissue Lyser LT (Qiagen) and bead-beating was applied for 3x30s with 40 oscillations per second. Subsequently, 250 µl of the sample were transferred to a new 2 ml tube and mixed by pipetting for 5min with 750 µl of TRIzol LS Reagent (Invitrogen). The RNA extraction was continued with the Direct-zol RNA Miniprep Plus kit (Zymo Research) according to the manufacturer's instructions. The cDNA synthesis was performed as described previously (Wylezich et al., 2018) with 2µg RNA input using cDNA-Synthesis System and Roche 400 µM Random-Hexamer-Primer (Roche). 100 ng of cDNA was fragmented to 350 bp using ultrasonication with M220 Focused-ultrasonicator (Covaris) and DNA libraries were generated with the TruSeq Nano DNA Library Prep kit (Illumina). DNA libraries were sequenced in paired-end mode with 2 × 151 cycles on the NextSeq 500 sequencing system (Illumina).

Workflow 6

M30-32: The salmon sample were thawed at 4°C for <1hr on arrival, supplemented with and centrifuged at 5000 rpm for 20 min at 4°C. The pellet was then transferred directly to a PowerBead tube from the Power Food kit and processed according to the standard kit protocol. Following DNA isolation, the DNA was quantified using the Eppendorf BioSpectrometer® and libraries were prepared using the Nextera XT Library prep kit (Illumina) accordingly to manufacturer's instructions. The libraries were sequenced on the Illumina NextSeq500 system kit (Illumina) in paired end mode with 2 x 150 cycles using standard sequencing protocols (Illumina).

Workflow 7

M07: 500 µl Solution PF1 (55°C) was added to the frozen spiked salmon sample and vortexed for 15 sec. The supernatant was transferred to a Power Bead Tubes Garnet 0.70 tube (Oxoid).

M10: The frozen spiked salmon sample was transferred to a Power Bead Tubes Garnet 0.70 tube (Oxoid). To collect any remaining sample material in the original tube, 500 µl Solution PF1 (55°C, solution PF1 precipitates when cold) (PowerFood Microbial DNA Isolation kit) was added to the tube that originally contained the salmon, vortexed for 10 sec, and the buffer transferred to the bead-beating tube as well. The beat-beating tube was placed on ice.

Both samples: The tube was inserted into the preheated (55°C) adapter of Tissue Lyser II and bead-beating was applied for 3 x 30 sec at 30 Hz. The tube was centrifuged at full speed for 1 min. Subsequently, supernatant was transferred to a new 2 ml tube. DNA extraction was performed using MoBio PowerFood Microbial DNA Isolation kit according to manufacturer's instructions using low-DNA microcentrifuge tube when eluting DNA from filter membrane. DNA underwent Nextera XT library preparation (Illumina) and the samples were sequenced on an Illumina Nextseq 500.

Workflow 8

M26: One milliliter of ice-cold buffer AL (Qiagen) was added to the frozen spiked salmon sample, transferred to a Lysing Matrix A tube (MP Biomedicals). The tube was inserted into the prechilled adaptor of Tissue Lyser LT (Qiagen) and bead-beating was applied for 3x30s with 40 oscillations per second. Subsequently, 500 µl of the sample were transferred to a new 2 ml tube. DNA extraction was performed using the QIAamp DNA Mini Kit according to manufacturer's instructions with 400 µl of the sample without the addition of buffer AL. 100 ng of DNA was fragmented to 350 bp using ultrasonication with M220 Focused-ultrasonicator (Covaris) and DNA libraries were generated with the TruSeq Nano DNA Library Prep kit (Illumina). DNA libraries were sequenced in paired-end mode with 2 × 151 cycles on the NextSeq 500 sequencing system (Illumina).

M36: The QIAamp DNA Mini Kit (Qiagen) was used for purification and it was carried out according to the manufacturer's instructions with modification. 720 µl of ALT buffer and 80 µl of proteinase K were added to the screw cap tube that contained the spiked salmon sample and mixed by inverting the tube 30×. The mixture was transferred a screw cap tube that contained equal amount of 1.0mm and 0.1mm sized silicon beads that was filled to the conical base of the screw cap tube. The tube was placed into FastPrep®-24 Classic Instrument (MP Biomedicals), for 1 minute, @ 4m/sec. Subsequently, ~800 µl of the supernatant (without salmon) was transferred to a new 2 ml tube and and incubated overnight at 56°C. After overnight incubation, the tube was placed at room temperature for 10 minutes prior to the addition of 8 µl of 100 mg/ml RNaseA (Qiagen). The tube was mixed and incubated for 10 minutes at room temperature. 800 µl of Buffer AL was added to the sample and mixed by inverting the tube 30× prior to incubation at 70°C for 10 minutes. After centrifugation for 10 minutes at 20,000g, 2 × 760 µl of supernatant was transferred to 2 × 1.5ml Eppendorf DNA LoBind Microcentrifuge Tube (Qiagen) that contained 380 µl of 96–100% molecular grade ethanol and mixed by inverting the tube 30×. 580 µl of supernatant was pipetted into the QIAamp Mini spin column and centrifuged at 6,000g for 1 minute. Flowthrough was discarded and the remaining supernatant was applied to the spin column. The tube was centrifuged at 6,000g for 1 minute and the flowthrough was discarded. The QIAamp Mini spin column was washed twice with 400 µl of Buffer AW1 and twice with of 400 µl Buffer AW2. After each wash, the column was centrifuged at 20,000g for 2 minutes and flowthrough was discarded. The spin column was placed into a clean 1.5ml Eppendorf tube and 40 µl of 60°C distilled water was added. The tube was incubated at room temperature for 5 minutes and then centrifuged at 6,000g for 3 minutes to elute DNA. The DNA concentration was quantified by Qubit® 3.0 Fluorometer (Thermo Fisher Scientific). Library preparation was performed according to Illumina's TruSeq Nano DNA Sample Preparation protocol. The samples were sheared on a Covaris S220 to ~450bp, following the manufacturer's recommendation, and uniquely tagged with Illumina's TruSeq HT DNA dual barcodes to enable library pooling for sequencing. Finished libraries were quantitated using Invitrogen's Picogreen assay and the average library size was determined on a Bioanalyzer 2100 (Agilent) or a Tapestation 4200 (Agilent). Library concentrations were then normalized to 4nM and validated by qPCR on a ViiA-7 real-time thermocycler (Applied Biosystems), using qPCR primers recommended in Illumina's qPCR protocol, and Illumina's PhiX control library as standard. The

libraries were then pooled at 3.5nM in batches of 10 samples and each pool was sequenced in 1 lane on an Illumina HiSeq2500 sequencer in rapid mode at a read-length of 250bp paired-end.

Workflow 9

M25: RNA was extracted from the entire spiked smoked salmon sample using the QIAamp Viral RNA kit as per the manufacturer's instructions. cDNA synthesis was achieved using 8µl extracted RNA as the input into the cDNA-Synthesis System (Roche) combined with 400 µM Random-Hexamer-Primer (Roche) and manufacturer's instructions were followed. The double-stranded cDNA was then diluted to 0.2 ng µl-1 with molecular biology grade water and 1 ng was used as the input to the NexteraXT library preparation kit (Illumina) and processed according to the manufacturer's instructions. The DNA library was then sequenced in paired-end mode with 2 × 151 cycles on the NextSeq 500 sequencing system (Illumina).

Workflow 10

M37: The spiked salmon sample was homogenized in PBS using ceramic beads and then centrifuged at 4,000 g for 10 min. 140 µl of the supernatant was collected for extraction with the Qiagen viral RNA mini kit according to the protocol but without addition of carrier RNA. The extracted RNA/DNA was eluted in 60 µl of nuclease-free water. 10 µl was used fo cDNA synthesis with the superscript IV (Thermo Fisher Scientific) using random hexamers. The reaction was further processed for double stranded cDNA synthesis (NEBNext® Ultra II Non-Directional RNA Second Strand Synthesis Module, NEB). The reaction was purified using AMPure XP beads (Beckman Coulter). DNA was quantified using the HS dsDNA Assay Kit with the Qubit (Thermo Fisher Scientific). The library was prepared using the Nextera XT Library prep kit (Illumina) and sequenced on the Illumina MiSeq.

Workflow 11

M08: Spiked salmon sample was homogenized in liquid nitrogen using a ceramic mortar and pestle followed by the gradual addition of 2.5 ml TE buffer. The homogenate was then centrifuged at 4,000 x g for 10 min and the supernatant was carefully collected. The supernatant was filtered on a 0.45-µm disc HPF Millex syringe filter (Millipore, Cork, Ireland) followed by nuclease treatment. Subsequently, 200 µl of the sample was transferred to a new tube and DNA was isolated using the QIAamp DNA Mini Kit (Qiagen, Hilden, Germany) according to manufacturer's instructions. Tag labeling and random amplification (SISPA) was performed as described previously (Allander et al., 2005; Blomström et al., 2010). Sequencing libraries were prepared using the Nextera XT DNA sample preparation kit (Illumina, San Diego, CA) according to the manufacturer's instructions. Library quantity and quality was validated and monitored by Agilent 2100 Bioanalyzer (Agilent Technologies, Santa Clara, CA), normalized by adjusting molar concentration to 4 nM with EB buffer (Qiagen), pooled and further prepared for sequencing according to the manufacturer's instructions (Illumina). Pooled libraries were sequenced using MiSeq Reagent Kit v3 in paired-end mode with 2 x 300 cycles on the Illumina MiSeq platform.

M11, M13: Spiked salmon sample was homogenized in liquid nitrogen using a ceramic mortar and pestle followed by the gradual addition of 2.5 ml TE buffer. The homogenate was then centrifuged at 4,000 x g for 10 min and the supernatant was carefully collected. Next, sample was subjected to three cycles of freeze-thaw method (M11: dry ice/100 oC; M13: dry ice/37 oC) and followed by DNA isolation using the QIAamp DNA Mini Kit (Qiagen, Hilden, Germany) according to manufacturer's instructions. Tag labeling and random amplification (SISPA) was performed as described previously (Allander et al., 2005; Blomström et al., 2010). Sequencing libraries were prepared using the Nextera XT DNA sample preparation kit (Illumina, San Diego, CA) according to the manufacturer's instructions. Library quantity and quality was validated and monitored by Agilent 2100 Bioanalyzer (Agilent Technologies, Santa Clara, CA), normalized by adjusting molar concentration to 4 nM with EB buffer (Qiagen), pooled and further prepared for sequencing according to the manufacturer's instructions (Illumina). Pooled libraries were sequenced using MiSeq Reagent Kit v3 in paired-end mode with 2 x 300 cycles on the Illumina MiSeq platform.

Workflow 12

M27: The DNA-based workflow as applied for processing of sample starts with a disintegration step using the Covaris cryoPREP CP02 as described in Wylezich et al. (2018). The DNA of the disintegrated samples was extracted with the QIAamp Mini DNA Kit (Qiagen) according to manufacturer's instructions. The library preparation starting with fragmentation was done as detailed described in Wylezich et al. (2018).

Workflow 13

M19: The frozen spiked salmon sample was transferred to a Power Bead Tubes Garnet 0.70 tube (Oxiod) and placed on ice. To collect any remaining sample material in the original tube, 1 ml Buffer AL (Qiagen) was added to the tube that originally contained the salmon, vortexed for 10 sec, and the buffer transferred to the bead-beating tube as well. The beat-beating tube was placed on ice.

M20: 900 µl Buffer AL (Qiagen) was added to the frozen spiked salmon sample and vortexed for 15 sec. The supernatant was transferred to a Power Bead Tubes Garnet 0.70 tube (Oxoid). The bead-beating tube was placed on ice.

Both samples: The bead beating tube was vortexed and inserted into the prechilled adapter of a Tissue Lyser II and bead-beating was applied for 3 x 30 sec at 30 Hz. The tube was centrifuged at full speed for 1 min at 4°C, and placed on ice. Subsequently, the RNA extraction was performed based on RNeasy Mini kit (Oxoid) with a TRIzol LS (Invitrogen) pre-treatment according to ("Harmonized Step-by-step Protocols" at https://www.compare-europe.eu/Project-organisation/Work-packages/Workpackage-2/Protocols-for-Sample-Processing-for-NGS). The cDNA synthesis was performed with 1µg RNA based on Roche cDNA-Synthesis system and Roche 400 µM Random-Hexamer-Primer (Roche) according to ("Harmonized Step-by-step Protocols" at https://www.compare-europe.eu/Project-organisation/Work-packages/Workpackage-2/Protocols-for-Sample-Processing-for-NGS). DNA underwent Nextera XT library preparation (Illumina) and the samples were sequenced on an Illumina Nextseq 500.

Workflow 14

M12: Spiked salmon sample was homogenized in liquid nitrogen using a ceramic mortar and pestle followed by the gradual addition of 2.5 ml TE buffer. The homogenate was then centrifuged at 4,000 x g for 10 min and the supernatant was carefully collected. The supernatant was filtered on a 0.45-µm disc HPF Millex syringe filter (Millipore, Cork, Ireland) followed by nuclease treatment. Subsequently, 200 µl of the sample was transferred to a new tube and mixed with 600 µl of TRIzol Reagent (Invitrogen, Carlsbad, CA), 160 µl of chloroform followed by RNeasy Mini Kit (Qiagen, Hilden, Germany) RNA isolation according to the manufacturer's instructions. The cDNA synthesis using SuperScript IV (Invitrogen, Carlsbad, CA), Tag labeling and random amplification (SISPA) was performed as described previously (Allander et al., 2005; Blomström et al., 2010). Sequencing libraries were prepared using the Nextera XT DNA sample preparation kit (Illumina, San Diego, CA) according to the manufacturer's instructions. Library quantity and quality was validated and monitored by Agilent 2100 Bioanalyzer (Agilent Technologies, Santa Clara, CA), normalized by adjusting molar concentration to 4 nM with EB buffer (Qiagen), pooled and further prepared for sequencing according to the manufacturer's instructions (Illumina). Pooled libraries were sequenced using MiSeq Reagent Kit v3 in paired-end mode with 2 x 300 cycles on the Illumina MiSeq platform.

Workflow 15

M28: The RNA-based workflow as exactly applied for processing of sample is detailed in Wylezich et al. (2018) and including sample disintegration using the cryoPREP CP02 device.
